# Prognostic Role of Serum Albumin in Predicting 30-Day Mortality in Patients with Infections in Emergency Department: A Prospective Study

**DOI:** 10.3390/jcm12103447

**Published:** 2023-05-13

**Authors:** Gianni Turcato, Arian Zaboli, Serena Sibilio, Massimiliano Fanni Canelles, Eleonora Rella, Alberto Giudiceandrea, Norbert Pfeifer, Francesco Brigo

**Affiliations:** 1 Department of Internal Medicine, Intermediate Care Unit, Hospital Alto Vicentino (AULSS-7), 36014 Santorso, Italy; gianni.turcato@yahoo.it; 2 Department of Emergency Medicine, Hospital of Merano-Meran (SABES-ASDAA), 39012 Merano-Meran, Italy; zaboliarian@gmail.com (A.Z.); serena.sibilio@sabes.it (S.S.); massimiliano.fannicanelles@sabes.it (M.F.C.); eleonora.rella@sabes.it (E.R.); alberto.giudiceandrea@sabes.it (A.G.); norbert.pfeifer@sabes.it (N.P.); 3 Lehrkrankenhaus der Paracelsus Medizinischen Privatuniversität, 5020 Salzburg, Austria; 4 Department of Neurology, Hospital of Merano-Meran (SABES-ASDAA), 39012 Merano-Meran, Italy

**Keywords:** albumin, infection, sepsis, septic shock, emergency department, serum albumin

## Abstract

Background: Infections in emergency departments (EDs) are insidious clinical conditions characterised by high rates of hospitalisation and mortality in the short-to-medium term. The serum albumin, recently demonstrated as a prognostic biomarker in septic patients in intensive care units, could be an early marker of severity upon arrival of infected patients in the ED. Aim: To confirm the possible prognostic role of the albumin concentration recorded upon arrival of patients with infection. Methods: A prospective single-centre study was performed in the ED of the General Hospital of Merano, Italy, between 1 January 2021 and 31 December 2021. All enrolled patients with infection were tested for serum albumin concentration. The primary outcome measure was 30-day mortality. The predictive role of albumin was assessed by logistic regression and decision tree analysis adjusted for Charlson comorbidity index, national early warning score, and sequential organ failure assessment (SOFA) score. Results: 962 patients with confirmed infection were enrolled. The median SOFA score was 1 (0–3) and the mean serum albumin level was 3.7 g/dL (SD 0.6). Moreover, 8.9% (86/962) of patients died within 30 days. Albumin was an independent risk factor for 30-day mortality with an adjusted hazard ratio of 3.767 (95% CI 2.192–6.437), *p* < 0.001. Decision tree analysis indicated that at low SOFA scores, albumin had a good predictive ability, indicating a progressive mortality risk reduction in concentrations above 2.75 g/dL (5.2%) and 3.52 g/dL (2%). Conclusions: Serum albumin levels at ED admission are predictive of 30-day mortality in infected patients, showing better predictive abilities in patients with low-to-medium SOFA scores.

## 1. Introduction

Infections are insidious and often life-threatening clinical conditions observed daily in emergency departments (EDs) [1]. In the United States, they account for approximately 850,000 ED visits per year, and their most severe forms, sepsis and septic shock, cause up to 35% of all in-hospital deaths [2,3].

While some infected patients in the ED initially present with clear signs of general tissue hypoperfusion and organ damage associated with sepsis or septic shock, the majority of infections are initially concealed by apparent clinical stability and undetectable, sudden deterioration with unpredictable, rapid evolution [2,4,5].

It is well known that the tools currently used in the clinical management of infections, such as blood tests, diagnostics, and scores, do not reach optimal predictive levels. Additionally, despite published guidelines, little guidance is available for the rapid recognition of evolutionary risk, especially for patients with initially less severe disease [5,6,7]. In fact, in contrast to septic states, where instability on arrival in the ED leads to the immediate initiation of treatment, the severity of infections without hypoperfusion may not be immediately identified, postponing potentially life-saving care [5,6,8].

Serum albumin concentration has recently been described as a predictive biomarker of mortality in septic patients admitted to intensive care units (ICUs) [9,10]. Low albumin levels are associated with high sepsis severity scores, high circulating levels of tumour necrosis factor (TNF), interleukin (IL)-1 and IL-6, and the presence of microcirculatory dysfunction seen in capillary leak syndrome, which is responsible for the poor prognosis of infections [11,12]. Serum albumin concentration is a possible early biomarker of microvascular changes, and thus indirectly of infection severity, which may make albumin an important prognostic element for infections [12].

The aim of this prospective study is to evaluate the possible prognostic role of the albumin concentration recorded in patients with an infection on arrival in the ED.

## 2. Methods

### 2.1. Study Design and Setting

This prospective observational cohort study included patients aged 18 and over with an infection and took place at the ED of the General Hospital of Merano (Italy, 53,000 admissions in 2021) between 1 January 2021 and 31 December 2021. The study was conducted in accordance with the Declaration of Helsinki and approved by the local ethics committee (approval number 94-2020).

### 2.2. Patients

All adult patients (aged 18 years and older) who presented to the ED with symptoms of a suspected infection were considered for enrolment in the study. The decision to consider the patient to have an infection and start the study protocol by performing a serum albumin test was made independently by the ED physician treating the patient. At the end of the clinical-instrumental management performed in the ED, the diagnosis of infection was confirmed. Otherwise, the patient was excluded from subsequent analyses. The other exclusion criteria were as follows: (a) Severe hepatic dysfunction with a total serum bilirubin level higher than 30.0 mg/dL; (b) AIDS or pregnancy; (c) administration of human albumin in the 3 weeks preceding the onset of infection; (d) admission to the ED within 15 days of a previous hospitalisation or operation; (e) presence of COVID-19 infection.

All patients enrolled in the study provided informed consent before starting the study protocol. If informed consent could not be provided, it was requested from the closest relative present. Failure to provide consent excluded the patient from the study.

### 2.3. Study Protocol and Data Collection

At the first assessment of the patient with suspected infection, a panel of blood tests was performed. The tests were as follows: Complete blood count with leucocyte differential, serum electrolyte levels, renal function, liver function, serum albumin, C-reactive protein (CRP), total bilirubin, coagulation status, and arterial blood gas.

For the determination of albumin levels, a 3 mL sample of peripheral venous blood was collected. After centrifugation, the serum was decanted and the albumin levels were measured by bromocresol green colourimetry using the Alinity ci-series (Abbott, IL, USA). The albumin levels were provided in g/dL.

Demographic and clinical data, including gender, age, medical history, systolic blood pressure, diastolic blood pressure, respiratory rate, heart rate, capillary oxygen saturation, and cognitive impairment were collected at the first physician’s evaluation in the ED. In addition, the Charlson comorbidity index (CCI), the national early warning score (NEWS), and the sequential organ failure assessment (SOFA) score were reported.

### 2.4. Outcome

The primary outcome of the study was death of the patient within 30 days of the first evaluation in the ED. Mortality was determined using death records or by contacting the registry office directly.

### 2.5. Statistical Analysis

Categorical variables were expressed as percentages and the number of events in the total, and univariate comparisons were performed with Fisher’s exact tests and chi-square tests. Continuous variables were expressed as mean and standard deviation (SD) or median and interquartile range, depending on the underlying distribution. Comparisons were performed with Student’s *t*-tests, Mann–Whitney, or Kruskal–Wallis tests where appropriate. To assess the discriminatory ability of albumin, we calculated the area under receiver operating characteristic (AUROC) of albumin in comparison with the most widely used multi-parameter scores (NEWS, CCI, SOFA) to predict 30-day mortality. To test the prognostic role of albumin and validate its effect on 30-day mortality, logistic regression models were developed using the CCI (a surrogate for medical history and comorbidity severity), NEWS (a surrogate for immediate urgency), and SOFA (a surrogate for prognostic severity) as possible clinical confounders. The independent association of albumin levels (for each 1 g/dL decrease) with mortality was reported as an adjusted odds ratio (OR) and 95% confidence interval (95% CI).

A decision tree analysis was performed with the same variables to assess the prognostic effect of albumin on 30-day mortality. Decision tree analyses are powerful data mining analyses that create a non-parametric supervised learning algorithm. The decision tree was developed using the chi-square automatic interaction detection (CHAID) technique [13]. This consists of a hierarchical tree structure comprising a root node, branches, internal nodes, and leaf nodes. At each classification level along the tree, the model identifies the most significant predictor with the chi-square test to subdivide the data interactively [13]. At the first level of subdivision, the node at the top of the hierarchy is the root node. Subsequent levels include parent nodes, which are subdivided into other nodes at lower levels. Leaf nodes, which are not further subdivided, identify subgroups of patients sharing the same risk [13]. To resolve any overfitting, a 10-fold cross-validation was used. The predictive performance of the decision tree for 30-day mortality was calculated by reporting the estimated corrected classifications.

All results were considered as statistically significant for *p*-values < 0.05. STATA 16.0 and SPSS statistical software packages were used for the analyses.

## 3. Results

**Figure 1:**
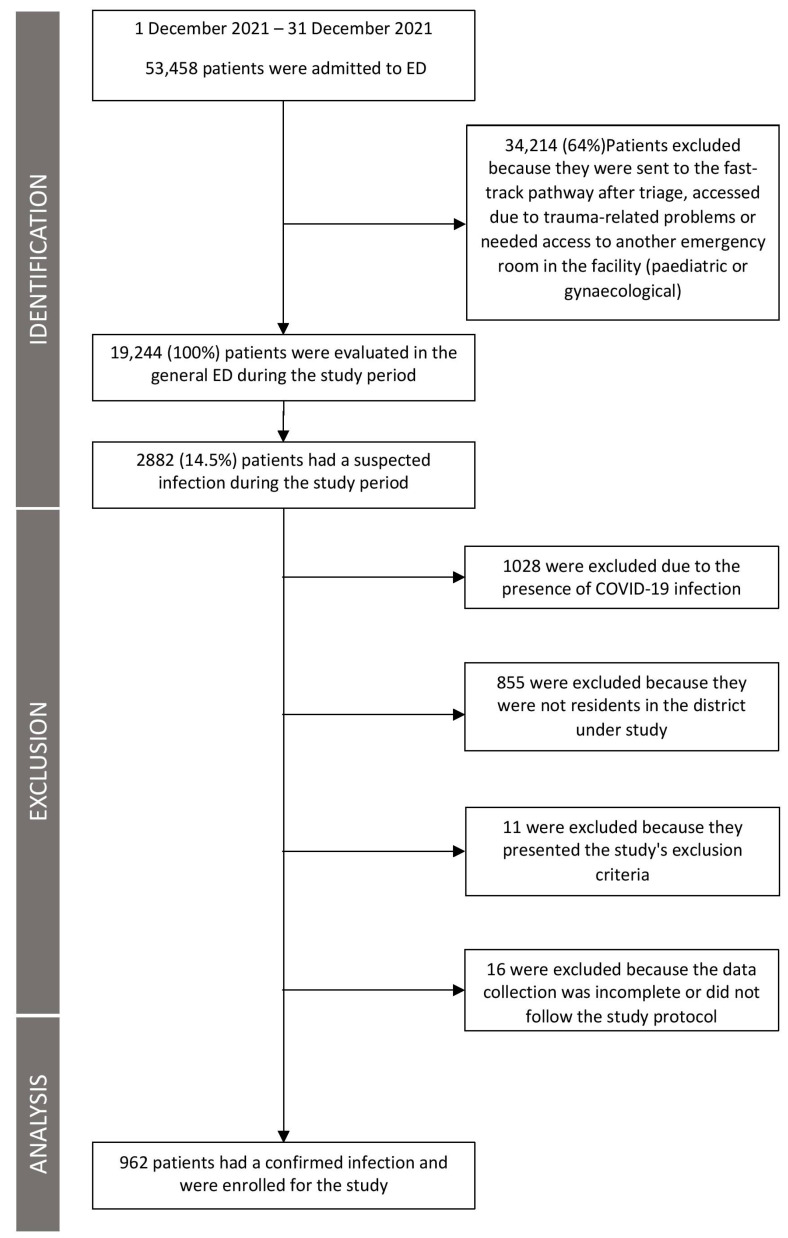
Flow chart of patients enrolled in the study.

Nine hundred and sixty two patients with confirmed infection were enrolled in the study (Figure 1). The median SOFA score recorded in the study population was 1 (0–3) and 47.7% (459/962) of the patients had a SOFA score of 2 or greater. The mean albumin level recorded on arrival in the ED was 3.7 g/dL (SD 0.6). The baseline characteristics of the patients are reported in Table 1.

Patients with an albumin level below the mean were older, had more comorbidities, and more altered vital signs on arrival in the ED. Specifically, patients with below-mean albumin had a median CCI of 5 (4–7) versus a median CCI of 2 (0–5) in patients with above-mean albumin (*p <* 0.001), a median NEWS of 4 (2–7) versus a median NEWS of 2 (1–4) (*p* > 0.001), and a median SOFA score of 3 (1–4) versus a median SOFA score of 1 (0–2) (*p <* 0.001).

The albumin level recorded at admission showed a significant correlation with CCI (Spearman’s rho −0.521, *p <* 0.001), SOFA score (Spearman’s rho −0.478, *p <* 0.001), and NEWS (Spearman’s rho −0.411, *p <* 0.001). Moreover, 8.9% (86/962) of the patients died within 30 days of ED evaluation. Their characteristics are listed in Table 2.

Patients who died within 30 days were older and had a higher rate of comorbidities. In particular, the mean CCI value in these patients was 6.3 (SD 2.1) versus a mean of 3.8 (SD 2.9) in the surviving patients (*p <* 0.001). NEWS and SOFA scores were also significantly higher in patients who died within 30 days.

The mean albumin level recorded at ED admission in patients who survived to 30 days was 3.8 g/dL (SD 0.5), whereas the mean in patients who died at 30 days was 3.1 g/dL (SD 0.5) (*p <* 0.001). Among patients with below-average albumin (<3.7 g/dL), 17.1% (80/467) died at 30 days compared to only 1.2% (6/495) of patients with above-average albumin (*p <* 0.001).

**Figure 2:**
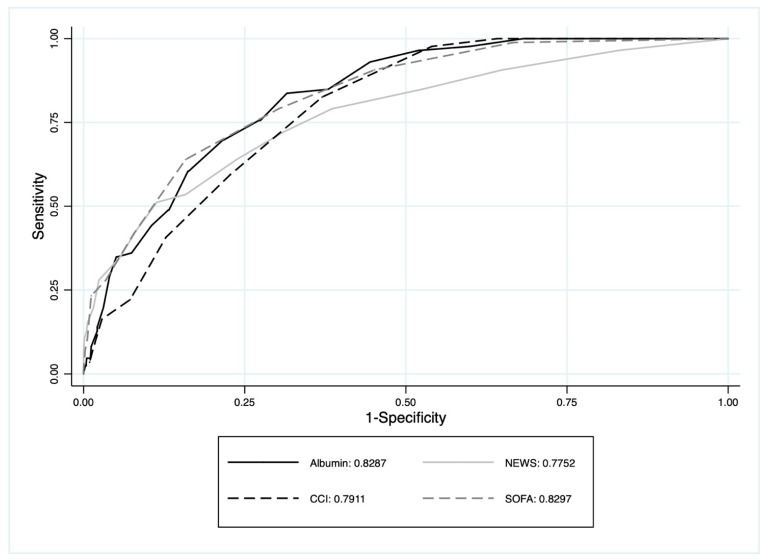
The area under the ROC curve of albumin, SOFA, NEWS, and CCI to predict 30-day mortality.

The multivariate model, which adjusted for anamnestic severity (CCI), urgency (NEWS), and prognostic severity (SOFA), revealed that serum albumin concentration was an independent predictor of mortality risk with an OR of 3.767 (95% CI 2.192–6.437) for each one-unit decrease. The discriminatory ability of albumin presented an AUROC of 0.821 (95% CI 0.782–0.860) (Figure 2). The AUROC of albumin (ROC: 0.821; 95% CI: 0.782–0.860), NEWS (ROC: 0.768; 95% CI: 0.711–0.826), CCI (ROC: 0.794; 95% CI: 0.754–0.833), and SOFA (ROC: 0.837; 95% CI: 0.798–0.876) showed that albumin alone performs as good as or even better than the other multi-parametric scores for predicting 30-day mortality in patients with infection in the ED (Figure 2).

The AUROC of the multivariate model was 0.893 (95% CI 0.863–0.923), with R-Square of 0.394 (*p <* 0.001).

**Figure 3:**
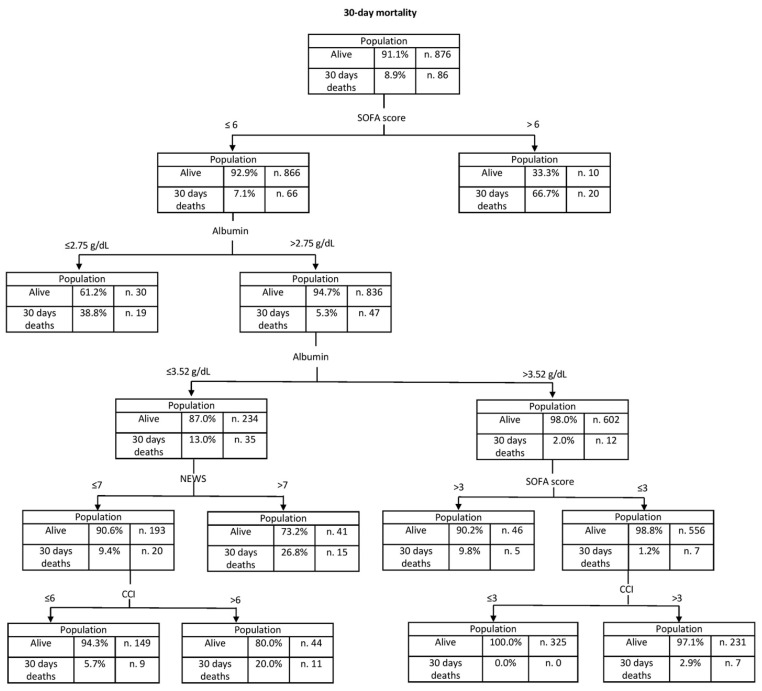
Decision tree model generated using the chi-squared automatic interaction detection method to illustrate the hierarchical association between albumin, NEWS, SOFA, CCI, and 30-day mortality. The rate of 30-day mortality for each node is reported.

The analysis of the decision tree, shown in Figure 3, confirmed the important role of albumin in the prognostic assessment of infected patients. Indeed, for patients with SOFA scores below 7, serum albumin levels above 2.75 g/dL and 3.52 g/dL indicate a progressive reduction in the risk of death within 30 days (5.2% and 2%, respectively). The decision tree showed a 98.9% ability to classify patients who survived to 30 days, and a 22.3% ability to classify patients who died within 30 days.

The predictive ability of albumin for 30-day mortality was found to be higher in patients with low SOFA scores (Table 3) with a preponderant role in patients who appear to be low-risk.

## 4. Discussion

Using a large cohort of patients who were admitted to the ED for an infection, the findings of this prospective study confirmed that the serum albumin concentration immediately recorded on ED admission is an independent risk factor for 30-day mortality (OR 3.767, 95% CI 2.192–6.437). Its predictive ability appeared to be higher in patients with lower SOFA scores at admission, which indicate a lower risk. Our innovative decision tree analysis appears to have confirmed this predictive role.

Previous reports have described the association between hypoalbuminemia and mortality in various medical conditions, such as stroke and myocardial infarction. However, the available data on albumin at the time of ED admission and sepsis outcomes are scarce, and almost absent on infections more generally [14,15]. These are often insidious conditions, where apparent clinical stability on arrival in the ED may conceal the microcirculatory alterations in tissues, which can lead to the organ dysfunction typical of sepsis [5,12]. Therefore, a flexible, rapid, inexpensive biomarker capable of integrating into the assessment of the infected patient and indicating potentially higher severity early after ED admission may be useful in the complex estimation of the infected patient’s prognosis.

The study was designed to cover previously highlighted gaps. The prospective study design, the collection of albumin at the first medical evaluation in the ED, and the cohort of patients with infections, not sepsis alone, are novelties provided by the study.

The mean serum albumin concentration recorded in patients who died within 30 days (3.1 g/dL, SD 0.5) was higher than the values reported in studies that only considered septic patients in the ICU, but in line with the results of more generalist studies conducted in the ED [10,12,16]. Ayranci et al., in a recent retrospective study on elderly patients in the ED, showed that the albumin level was 3.97 g/dL (3.55–4.25) in patients who survived versus 3.36 g/dL (2.86–3.92) in patients who died in hospital [17]. In ICU studies on septic patients, the albumin levels recorded on arrival in the ICU were generally below 3 g/dL [9,10].

Even after adjustment for baseline disease condition (CCI), severity at presentation (NEWS), and short-term prognosis (SOFA), albumin was a significant independent risk factor for 30-day mortality with an OR of 3.767 (95% CI 2.192–6.437). The confounding variables used to adjust the association of albumin with the outcome were chosen a priori. However, choosing known predictive tools for history, priority, and prognosis can provide an overall idea of the severity of the patient’s condition. Arneu-Barrès et al., in a retrospective study of 235 elderly patients with sepsis in the ED, demonstrated that an albumin level under 2.6 g/dL had an odds ratio (OR) of 3.26 (95% CI 1.12–9.41, *p* = 0.029) for the risk of death at 30 days [18].

In a secondary analysis of data from a large study of all patients with acute medical conditions admitted to a hospital, Jellinge et al. suggested hypoalbuminemia (with a cut-off below 3.5 g/dL) as an independent predictor of 30-day all-cause mortality with an OR of 1.95 (95% CI 1.31–2.90) adjusted for sex, age, CCI, and Worthing physiological score [19]. The authors suggested the limitation of measuring albumin levels at the time of admission only [19]. This finding is also confirmed by a retrospective study including all hospitalised patients, showing that as the albumin value decreased, both the risk of in-hospital mortality and the risk of 1-year mortality increased. For albumin values ranging from 3.0 g/dL to 3.4 g/dL, the OR for the risk of in-hospital death was 2.90 (95% CI 1.94–4.32) and for death at 1 year, the HR was 2.29 (95% CI 2.03–2.58). Risks were even higher for albumin values lower than 3.0 g/dL to 3.4 g/dL [20]. According to Yin et al., in 116 septic patients admitted to the ICU, an albumin level below 2.92 g/dL was an independent risk factor for 28-day mortality with a HR of 2.19 (95% CI 1.02–8.71, *p* = 0.045) adjusted for APACHE score, acute kidney injury, blood glucose levels, SOFA score, and use of invasive ventilation [21].

Finally, one of the important innovations in our study is the use of decision tree analysis, a powerful statistical technique that succeeds in overcoming the inherent difficulties of classical multivariate analyses. While high SOFA scores are sufficient to classify patients with poor prognosis, at low-medium SOFA scores, albumin may play an important role in the prognostic evaluation of infected patients. In this large population of patients admitted to the ED, the mortality risk is reduced for albumin levels above 2.75 g/dL and minimised for levels above 3.52 g/dL. Although no similar analyses are available, an age-stratified analysis by Frenkel et al. showed that the association of low albumin with mortality was stronger in younger patients admitted to the ICU, thus hypothetically overlapping in prognostic severity [9].

Confirmation of our results might suggest that a decrease in albumin, which has excellent predictive abilities regarding mortality in patients classified as lower risk (SOFA 0–1, 2–3), is a reason to suspect the presence of infection-induced microvascular changes. This is particularly important for patients who appear clinically stable on arrival in the ED despite the onset of capillary leak syndrome, which worsens the prognosis of septic patients. Due to the preliminary nature of our findings, further multicenter studies are required to define which albumin cut-offs should be used in clinical practice.

The study has few limitations. First, the single-centre design subjects it to the inherent biases of these studies. Second, anthropometric characteristics (weight/height) were not recorded as possible indicators of malnutrition, which could alter basal albumin levels. The European Society for Clinical Nutrition and Metabolism (ESPEN) guidelines recommend viewing albumin as a marker for the severity of an illness rather than nutritional status [22]. Third, therapeutic interventions used after evaluation in the ED that may have impacted the primary outcome were not considered. Fourth, the decision to recruit patients and classify them as possibly infected was made by the ED physician, although there are currently no clear criteria to define patients with suspected infection. It was, therefore, not possible to adopt a prespecified definition of infection, also due to the high heterogeneity present in this category of patients. Considering only patients with prespecified altered laboratory tests would have automatically led to the exclusion of a certain number of patients who have an infection. For example, including only patients with a positive blood culture would have excluded those with a viral infection. Conversely, patients with flu-like symptoms or a trivial urinary tract infection could have been less likely to undergo blood culture examinations, with the risk of under ascertainment [1,6]. A last limitation of our study is that the percentage of septic patients in the study population was high; this may be related to the non-unique definition of suspected infection [23].

## 5. Conclusions

The serum albumin level recorded on arrival of the infected patient in the ED was a significant independent predictor of 30-day mortality, even after adjusting for other important prognostic indicators. Albumin had a stronger predictive ability in apparently less severe patients, suggesting a role as a rapid marker of the early microvascular changes associated with infection mortality.

## Figures and Tables

**Table 1:** Demographic and clinical characteristics of the patients enrolled in the study divided according to the mean albumin value recorded in the patients.

Variables	Albumin < 3.7 g/dL	Albumin ≥ 3.7 g/dL	*p*-Value
Patients, n (%)	467 (48.5)	476 (51.5)	
Sex, n (%)			0.361
Female	190 (40.7)	216 (43.6)	
Male	277 (59.3)	279 (56.4)	
Age, years, mean (SD)	77.6 (13.3)	58.2 (21.5)	<0.001
Baseline characteristics, n (%)			
Ischaemic heart disease	95 (20.4)	53 (11.1)	<0.001
Hypertension	344 (73.7)	187 (39.3)	<0.001
Atrial fibrillation	123 (26.3)	49 (10.3)	<0.001
Diabetes	83 (17.8)	41 (8.6)	<0.001
Chronic kidney failure	88 (18.9)	27 (5.7)	<0.001
Chronic heart failure	112 (24.1)	43 (9.0)	<0.001
Chronic respiratory failure	65 (14.0)	34 (7.1)	0.001
Pulmonary embolism	17 (3.7)	13 (2.8)	0.46
Hepatopathy	21 (4.6)	6 (1.3)	0.003
Stroke or transient ischemic attack	45 (9.6)	17 (3.6)	0.002
Active tumour	65 (14.0)	34 (7.1)	<0.001
Dementia	108 (23.2)	36 (7.6)	<0.001
Neurological degenerative pathology	56 (12.1)	33 (6.9)	0.01
CCI, mean (SD)	5.3 (2.4)	2.8 (2.8)	<0.001
Vital parameters			
Systolic blood pressure, mean (SD)	120.8 (25.6)	134.5 (23.1)	<0.001
Diastolic blood pressure, mean (SD)	69.9 (14.2)	77.6 (12.1)	<0.001
Respiratory rate, median (IQR)	20 (16–27)	18 (16–22)	0.026
Heart rate, median (IQR)	98 (83–110)	94 (80–110)	0.006
Peripheral oxygen saturation, median (IQR)	95 (92-97)	97 (95-98)	0.001
Temperature, mean (SD)	37.8 (1.1)	37.8 (1.1)	0.476
NEWS, median (IQR)	4 (2–7)	2 (1–4)	<0.001
SOFA, median (IQR)	3 (1–4)	1 (0–2)	<0.001

**Table 2:** Demographic and clinical characteristics of patients enrolled in the study divided according to patients who died or survived at 30 days after ED evaluation.

Variables	Alive at 30 Days	Dead at 30 Days	*p*-Value
Patients, n (%)	876 (91.1)	86 (8.9)	
Albumin, g/dL, mean (SD)	3.8 (0.5)	3.1 (0.5)	<0.001
Albumin, n (%)			<0.001
<3.7 g/dL	387 (44.2)	80 (93)	
≥3.7 g/dL	489 (55.8)	6 (7)	
Sex, n (%)			0.086
Female	362 (41.3)	44 (51.2)	
Male	514 (58.7)	42 (48.8)	
Age, years, mean (SD)	65.9 (20.5)	84.7 (8.4)	<0.001
Baseline characteristics, n (%)			
Ischaemic heart disease	126 (14.4)	24 (27.9)	0.003
Hypertension	466 (53.2)	73 (84.9)	<0.001
Atrial fibrillation	143 (16.3)	31 (36.0)	<0.001
Diabetes	104 (11.9)	21 (24.4)	0.002
Chronic kidney failure	90 (10.3)	26 (30.2)	<0.001
Chronic heart failure	135 (15.4)	23 (26.7)	0.009
Chronic respiratory failure	90 (10.3)	11 (12.8)	0.461
Pulmonary embolism	28 (3.2)	3 (3.5)	0.753
Hepatopathy	24 (2.8)	3 (3.5)	1
Stroke or transient ischemic attack	52 (6.0)	10 (11.6)	0.063
Active tumour	87 (9.9)	14 (16.3)	0.094
Dementia	106 (12.1)	40 (46.5)	<0.001
Neurological degenerative pathology	74 (8.5)	16 (18.6)	0.006
CCI, mean (SD)	3.8 (2.9)	6.3 (2.1)	<0.001
Vital parameters			
Systolic blood pressure, mean (SD)	129.3 (24.4)	109.2 (27.1)	<0.001
Diastolic blood pressure, mean (SD)	74.9 (13.2)	64.5 (14.8)	<0.001
Respiratory rate, median (IQR)	18 (16–24)	25 (18–30)	<0.001
Heart rate, median (IQR)	95 (80–110)	103 (87–120)	0.003
Peripheral oxygen saturation, median (IQR)	96 (94–98)	93 (89–96)	<0.001
Temperature, mean (SD)	37.8 (1.1)	37.6 (1.3)	0.052
NEWS, median (IQR)	3 (1–5)	8 (4–11)	<0.001
SOFA, median (IQR)	1 (0–3)	4 (3–6)	<0.001

**Table 3:** Discriminatory ability of albumin recorded at entry in the different groups of patients classified according to SOFA score. The ORs of albumin (for decrements of one unit in g/dL) obtained by multivariate analysis adjusted for CCI, NEWS, and SOFA in the different patient categories are reported.

SOFA Categories	Patients, n (%)	Albumin, Mean (SD)	AUROC (95% CI)	OR (95% CI)	*p*-Value
0–1	502 (52.2)	3.9 (0.5)	0.851 (0.746–0.957)	8.315 (2.077–33.287)	0.003
2–3	271 (28.2)	3.6 (0.5)	0.760 (0.665–0.855)	4.270 (1.762–10.347)	0.001
>4	189 (19.6)	3.3 (0.5)	0.674 (0.593–0.755)	2.354 (1.072–5.172)	0.033

## Data Availability

Data available on request due to privacy/ethical restrictions.
